# Multimodal Imaging of Subfoveal Pachydrusen Containing a Blood Flow Signal

**DOI:** 10.1155/2022/5680913

**Published:** 2022-06-08

**Authors:** Naoko Ishiguro, Takaaki Hayashi, Yoshiko Yamawaki, Kei Mizobuchi, Tsutomu Yasukawa, Shigeru Honda, Tadashi Nakano

**Affiliations:** ^1^Department of Ophthalmology, The Jikei University School of Medicine, Tokyo, Japan; ^2^Department of Ophthalmology, Katsushika Medical Center, The Jikei University School of Medicine, Tokyo, Japan; ^3^Department of Ophthalmology and Visual Science, Nagoya City University Graduate School of Medical Sciences, Aichi, Japan; ^4^Department of Ophthalmology and Visual Sciences, Osaka City University Graduate School of Medicine, Osaka, Japan

## Abstract

Individuals with pachydrusen, larger than 125 *μ*m, have a significantly thicker choroid than do those with soft drusen or reticular pseudodrusen. Little is known about cases of abnormal blood flow within pachydrusen. The purpose of this report was to demonstrate a blood flow signal within pachydrusen using optical coherence tomography (OCT) angiography. A 76-year-old Japanese woman presented with innumerable drusen/pachydrusen in both posterior poles. Her visual acuity was good. OCT showed subfoveal pachydrusen in the left eye, but no exudative changes. The subfoveal choroidal thickness was increased to 274 *μ*m in the left eye. OCT angiography revealed a blood flow signal within the pachydrusen. However, fluorescein and indocyanine green angiographies indicated no abnormal hyperfluorescent lesion in the macula of the left eye. During the 13-month follow-up, the blood flow signal in OCT angiography did not change in diameter, and no exudative change was observed. The blood flow signal may have properties of capillary blood vessels derived from the choriocapillaris, rather than angiogenic vessels from choroidal neovascularization or polypoidal choroidal vasculopathy/aneurysmal type 1 neovascularization.

## 1. Introduction

Drusen are the accumulation of yellow deposits between the retinal pigment epithelium (RPE) and Bruch's membrane due to abnormalities of the RPE. Soft drusen, which are an essential precursor of age-related macular degeneration, are defined as drusen with a diameter of ≥63 *μ*m or more [1]. Cases of soft drusen are often accompanied by thinning of the choroid. In 2018, Spaide [2] reported relatively large (≥125 *μ*m) pachydrusen with choroidal thickening, and Cheung et al. [3] demonstrated that patients with pachydrusen had a significantly thicker choroid than did those with soft drusen or reticular pseudodrusen. To the best of our knowledge, there are no reported cases of a blood flow signal within the pachydrusen in the absence of exudative changes as indicated by optical coherence tomography (OCT) angiography (OCTA). Here, we report a case of a patient in whom a blood flow signal was detected within subfoveal pachydrusen using OCTA.

## 2. Case Presentation

**Figure 1:**
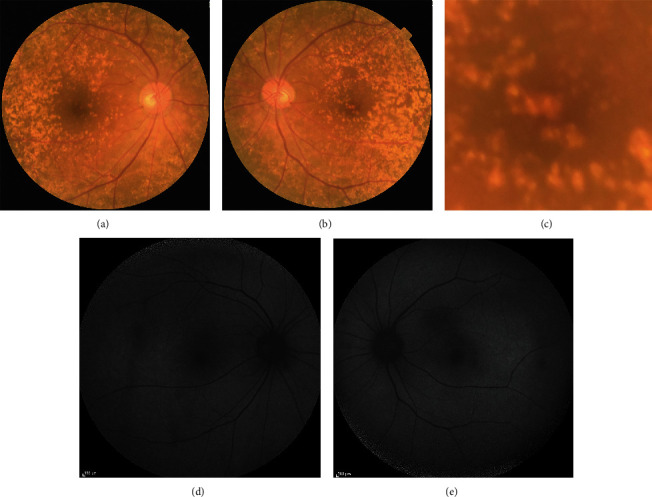
Color fundus photographs of the right (a) and left (b) eyes and the left macula (c). Fundus autofluorescence of the right (d) and left (e) eyes.

**Figure 2:**
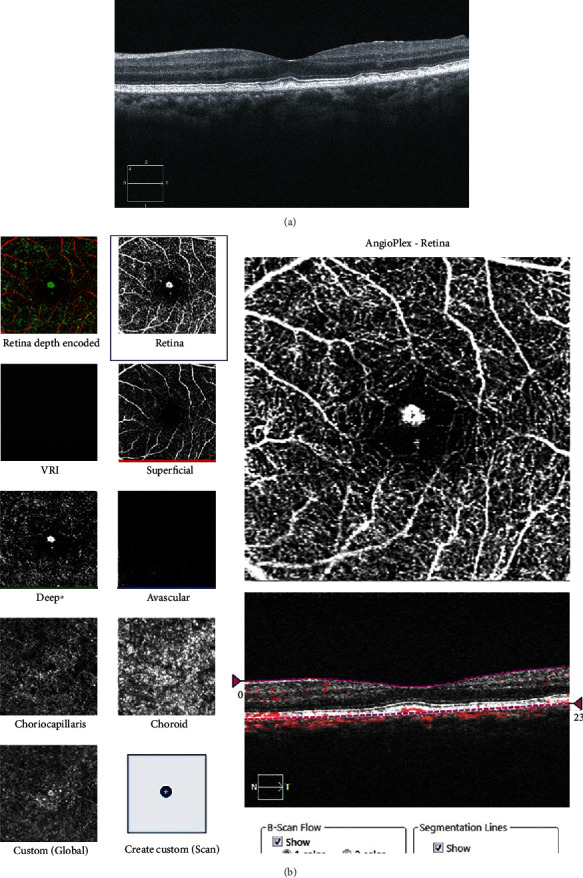
Horizontal optical coherence tomography (OCT) B-scan of the left eye (a) and OCT angiography of the left eye (b).

**Figure 3:**
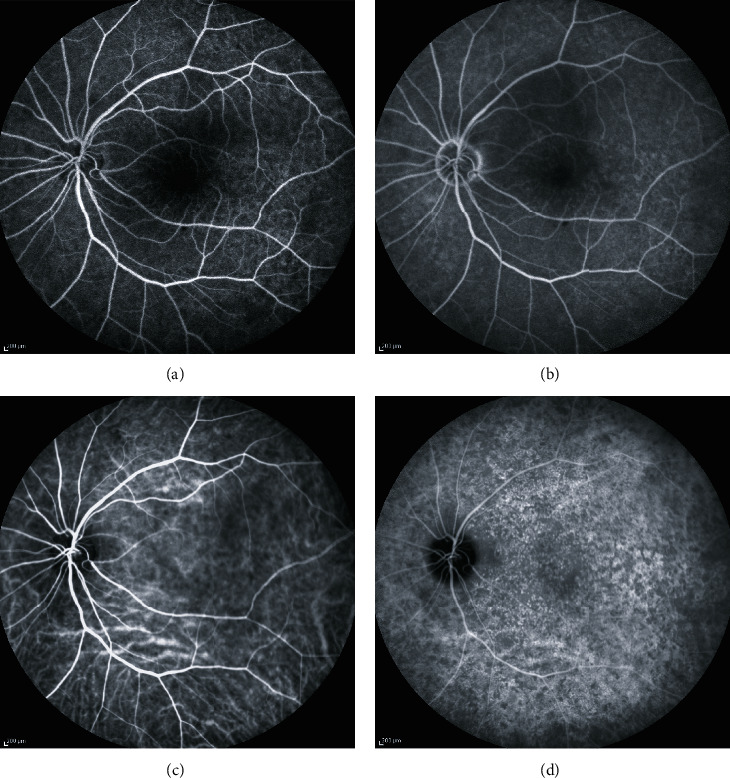
Fluorescein angiograms of the early (a) and late (b) phases in the left eye. Indocyanine green angiograms of the early (c) and late (d) phases in the left eye.

A 76-year-old Japanese female patient presented with blurred vision in her left eye. At presentation, her decimal best-corrected visual acuity was 1.0 (Snellen equivalent 20/20) in the right eye and 1.5 (Snellen equivalent 20/13) in the left eye with +3.00 diopter sphere, and her intraocular pressure was 15 mmHg in the right and 18 mmHg in the left eyes. There were no remarkable findings in the anterior segment and media, aside from mild cataracts in both eyes. On fundoscopy, innumerable drusen/pachydrusen were found in the posterior poles of both eyes (Figures 1(a) and 1(b)). Pachydrusen was found in the fovea of the left eye (Figure 1(c)). Fundus autofluorescence imaging using a confocal scanning laser scanning ophthalmoscope (Spectralis HRA, Heidelberg Engineering, Heidelberg, Germany) showed autofluorescence of the drusen on the temporal side of the macula of both eyes (Figures 1(d) and 1(e)). OCT (Cirrus 5000 Carl Zeiss Meditec, Dublin, CA) showed that the pachydrusen located at the RPE level were subfoveal in the left eye (Figure 2(a)). OCTA identified a blood flow signal in the subfoveal pachydrusen (Figure 2(b)). The horizontal diameter of the flow signal was 208 *μ*m. Fluorescein angiography (Figures 3(a) and 3(b)) and indocyanine green angiography (Figures 3(c) and 3(d)) revealed no hyperfluorescence/leakage, abnormal vasculature, vascular network, or polypoidal/aneurysmal lesions at the macula in the left eye in the early (Figures 3(a) and 3(c)) to late phase images (Figures 3(b) and 3(d)).

**Figure 4:**
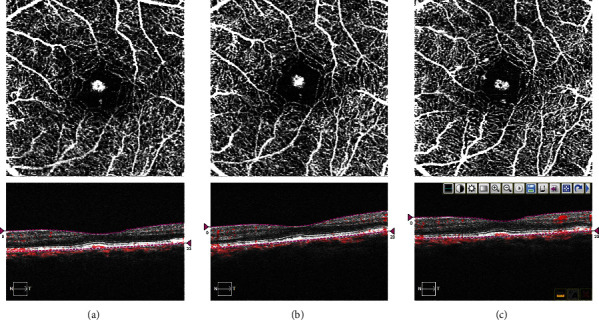
Optical coherence tomography angiography at baseline (a), 1 month later (b), and 13 months later (c) in the left eye.

**Figure 5:**
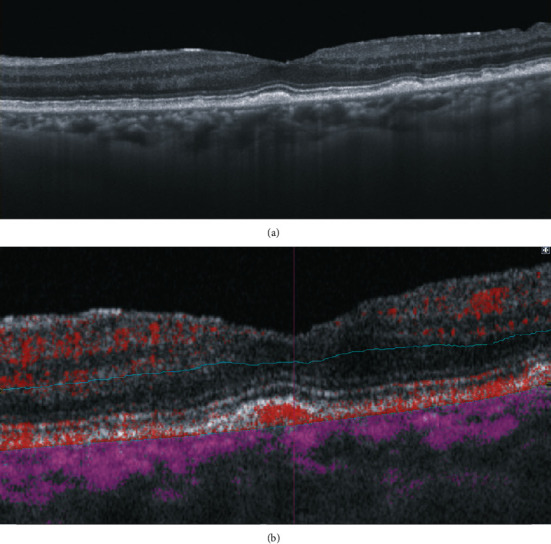
Horizontal swept-source optical coherence tomography (SS-OCT) B-scan (a) and horizontal B-scan SS-OCT angiography (b) at 13 months after presentation in the left macula.

**Figure 6:**
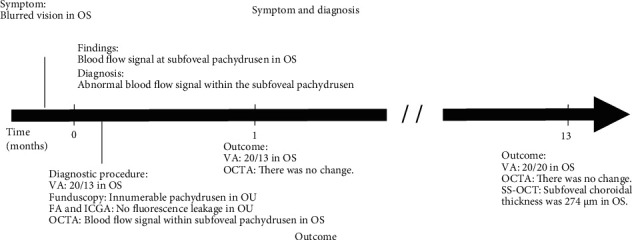
Clinical timeline of the female patient. OU: both eyes; FA: fluorescein angiography; ICGA: indocyanine green angiography; OCT: optical coherence tomography; OCTA: OCT angiography; SS-OCT: swept source OCT; VA: corrected visual acuity.

One month after presentation, her decimal best-corrected visual acuity was 1.5 in the left eye, and the size of the flow signal remained unchanged (Figures 4(a) and 4(b)). During the 13-month follow-up, the blood flow signal in OCTA did not change in horizontal diameter, and no exudative changes were observed (Figure 4(c)). At 13 months after presentation, her decimal best-corrected visual acuity was 1.0 in the left eye. On swept-source OCT (Triton, Topcon, Tokyo, Japan), subfoveal pachydrusen were clearly observed between the RPE and Bruch's membrane (Figure 5(a)), and the blood flow signal was detected within the pachydrusen (Figure 5(b)). The subfoveal choroidal thickness was 274 *μ*m. Clinical timeline of the patient is described in Figure 6.

## 3. Discussion

In this report, we describe a female patient in whom a blood flow signal was detected within the subfoveal pachydrusen on OCTA.

A Korean population-based cohort study on choroidal thickness in Asia demonstrated that the mean subfoveal choroidal thickness of 255 older people aged 65-99 years (mean age, 76.6 years) was 182 *μ*m [4]. In our patient, the subfoveal choroidal thickness was increased to 274 *μ*m. Recently, Cheung et al. investigated the association between choroidal thickness and drusen subtypes in Asian and white populations [3]. The Asian population included 145 older patients (mean age, 71.6 years) with drusen; the mean subfoveal choroidal thickness was significantly thicker in the pachydrusen group (*n* = 37, 265.5 *μ*m) than in the soft drusen group (*n* = 83, 218.0 *μ*m) and the pseudodrusen group (*n* = 25, 151.4 *μ*m). Similar outcomes were reported in a study of older Japanese patients [5]. Interestingly, in the Asian cohort [3], patients with polypoidal choroidal vasculopathy (also known as aneurysmal type 1 neovascularization), a pachychoroid spectrum disease [6], were more likely to have a thicker choroid and pachydrusen.

We believe that the blood flow signal (Figures 2, 4, and 5) in our patient originated from the choriocapillaris (or less likely from choroidal vessels directly). However, the signal was distinct from that of typical angiogenic vessels from choroidal neovascularization or a polypoidal/aneurysmal lesion in polypoidal choroidal vasculopathy because of the absence of fluorescein leakage in the fluorescein angiograms (Figure 3), vascular network, and polypoidal/aneurysmal vasculature on the indocyanine green angiograms (Figure 3) and any exudative changes on OCT (Figures 2 and 5). Thus, our multimodal imaging data demonstrated that the blood flow signal may have properties of capillary blood vessels containing endothelial cells, such as retinal capillaries. Although no apparent break in Bruch's membrane was observed on OCT (Figures 2 and 5), the blood flow would penetrate Bruch's membrane from the choriocapillaris and may have been associated with vulnerability of Bruch's membrane due to aging and/or the formation of pachydrusen. However, it cannot be denied that the flow signal may have been an indication of very early stage choroidal neovascularization or polypoidal choroidal vasculopathy. A recent retrospective cross-sectional study using OCTA has demonstrated that called vascularized drusen (presence of flow within the drusen) were seen in 5.5 percent of 128 patients with intermediate nonexudative age-related macular degeneration [7]. In that study [7], the vascularized drusen exhibited a uniform sub-RPE hyperreflectivity in OCT and lacked neovascular nature in fluorescein angiography. On the other hand, it was not mentioned whether pachydrusen patients are included. The subsequent consecutive study has revealed that 2 eyes with the vascularized drusen developed CNV during 24-month follow up [8].

In this report, we observed a blood flow signal within the subfoveal pachydrusen, but the actual cause remains unclear. Continued follow-up will be required to assess both OCT and OCTA findings and to elucidate the mechanism of development of the flow signal.

## Data Availability

All data studied in this case report are included in this report.
